# Human urinary kallidinogenase in acute ischemic stroke: A single‐arm, multicenter, phase IV study (RESK study)

**DOI:** 10.1111/cns.13724

**Published:** 2021-09-12

**Authors:** Jun Ni, Ming Yao, Li‐Hua Wang, Ming Yu, Run‐Hui Li, Li‐Hong Zhao, Jia‐Chun Wang, Yin‐Zhou Wang, Xin Wang, Hai‐Qing Song, Ben‐Yan Luo, Jia‐Wei Wang, Yi‐Ning Huang, Li‐Ying Cui, for the RESK Investigators, Li‐Ying Cui, Jun Ni, Ming Yao, Run‐Hui Li, Li‐Hong Zhao, Li‐Hua Wang, Jia‐Chun Wang, Ming Yu, Yan‐Lei Hao, Xin‐Ping Liu, Jie Lin, Bao‐Lin Shi, Long‐Xuan Li, Shi‐Ze Li, Chao‐Dong Zhang, Xiu‐E Wei, Ya‐Fen Wei, Guang‐Hua Li, Ben‐Yan Luo, Jian‐Jun Zhang, Li‐Yun Wang, Ying‐Qi Zhou, Xin‐Yi Li, Gao‐Hua Li, Jun Chen, Yin‐Zhou Wang, Yan Wei, Ji‐Hua Chen, Wei‐Min Liu, Qing‐Ke Bai, Jian‐Ping Niu, Shi‐Xiang Kuang, Mei Dong, Shi‐Ping Li, Xin‐Tong Liu, Xiang Gao, Yan‐Song Li, Kai Wang, Wei‐Min Xiao, Hai‐Qing Song, Guo‐Hua Zhao, Xing‐Yue Hu, Guo‐Fang Chen, Bo Li, Kan Ouyang, Xin Wang, Fu‐Ming Shi, Yu‐Wu Zhao, Xiao‐Gang Li, Dan‐Hong Wu, Qiang Li, Jing‐Bo Zhang, Guo‐En Yao, Ding Qin, Gang Li, Zheng‐Qi Lu, Qian Hou, Guang Huang, Xiao‐Juan Lin, Yi‐Ning Huang, Jun Peng, Xiao‐Kun Qi, Jia‐Wei Wang, Qi‐Dong Chen, Qiang Ma, Ping‐Yi Xu, Jia‐Wei Wang, Shu‐Yun Luo

**Affiliations:** ^1^ Department of Neurology State Key Laboratory of Complex Severe and Rare Diseases Peking Union Medical College Hospital Chinese Academy of Medical Sciences and Peking Union Medical College Beijing China; ^2^ Department of Neurology The Second Affiliated Hospital of Harbin Medical University Harbin China; ^3^ Department of Neurology The Affiliated Hospital of Jiangsu University Zhenjiang China; ^4^ Department of Neurology Central Hospital Affiliated to Shenyang Medical College Shenyang China; ^5^ Department of Neurology Dandong People's Hospital Dandong China; ^6^ Department of Neurology No. 1 Hospital of Harbin Harbin China; ^7^ Department of Neurology Fujian Provincial Hospital Fuzhou China; ^8^ Department of Neurology Zhongshan Hospital Fudan University Shanghai China; ^9^ Department of Neurology Xuanwu Hospital Capital Medical University Beijing China; ^10^ Department of Neurology The First Affiliated Hospital Zhejiang University School of Medicine Hangzhou China; ^11^ Department of Neurology Beijing Tongren Hospital Capital Medical University Beijing China; ^12^ Department of Neurology Peking University First Hospital Beijing China

**Keywords:** acute ischemic stroke, clinical trial, efficacy, human urinary kallidinogenase, safety

## Abstract

**Aims:**

Human urinary kallidinogenase (HUK) has shown favorable efficacies in acute ischemic stroke (AIS) treatment. We sought confirmation of the safety and efficacy of HUK for AIS in a large population.

**Methods:**

RESK study enrolled patients with AIS of anterior circulation to receive HUK infusion. The primary endpoint was the incidence of treatment‐emergent adverse events (AEs). Secondary endpoints assessed neurological and functional improvements and stroke recurrent rate.

**Results:**

Of 1206 eligible patients, 1202 patients received at least one dose of HUK infusion and 983 (81.5%) completed the study. The incidence of treatment‐emergent AEs and serious AEs were 55.99% and 2.41%, respectively. Pre‐specified AEs of special interest occurred in 21.71% of patients, but the majority were mild and unrelated to therapy. Hypertension, age, treatment time, and drug combination were identified to be associated with drug‐related blood pressure reduction. Neurological and functional evaluations revealed favorable outcomes from baseline to post‐treatment assessment. The cumulative recurrence rate of stroke was 2.50% during the 90‐day assessment.

**Conclusion:**

HUK had an acceptable safety and tolerability profile in AIS patients. Besides, HUK demonstrated the neurological and functional improvements in AIS, further confirming its clinical efficacy in a real‐world large population.

## Graphical abstract

In the prospective, multicenter, phase IV study (RESK study), eligible patients with acute ischemic stroke (AIS) received human urinary kallidinogenase (HUK) infusion. The results demonstrated that HUK had an acceptable safety and tolerability profile in AIS patients. Besides, HUK demonstrated the neurological and functional improvements in AIS, further confirming its clinical efficacy in a real‐world large population.

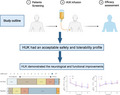

## 1 INTRODUCTION

Stroke represents a common and serious global healthcare problem.^1^ Acute ischemic stroke (AIS) is the most common subtype of stroke, accounting for 60%–80% of patients.^2^ Although stroke mortality has decreased worldwide in the past two decades, the overall global burden of stroke is great and increasing due to the aging population, particularly in developing countries.^3^ Any timely and efficient therapeutic strategies for AIS are the priority.

Although multiple treatment options for AIS have been provided, the optimal regime remains controversial.^4^ The conventional treatment is usually dissatisfactory.^5^ Recombinant tissue plasminogen activator as the standard treatment for AIS still has well‐recognized limitations.^6^ Besides, explorations on the acute phase of stroke treatment have mostly failed, including cerebral protection and collateral circulation improvement.^7^, ^8^ Accordingly, alternative options for AIS treatment remain the focus of global medical attention.

Recent evidence has convincingly established that the sufficiency of collateral circulation acts as a key factor influencing the likelihood of successful reperfusion and favorable outcome during conventional treatment.^9^, ^10^ Agents that augment collateral perfusion as an adjunctive strategy would be a valuable asset for AIS patients.^9^ Human urinary kallidinogenase (HUK) is a glycoprotein, which has been approved for stroke treatment.^11^ Notably, pharmacological evidence has proved that HUK enhances collateral circulation, cerebral blood flow, angiogenesis, and cerebral perfusion.^12^, ^13^ Previous trials have shown its potential efficacy in stroke treatment.^11^, ^14^ Although HUK is generally well tolerated, adverse events (AEs) related to HUK, especially blood pressure (BP) reduction, are still a question worthy of attention.^15^ However, recent results were from several small‐scale trials, and the evidence for the safety of HUK is still limited.

Therefore, the primary purpose of the RESK study was to evaluate the safety of HUK in patients with AIS, providing more evidence for its clinical application, as well as the risk management and control of medical institutions. The secondary objective was to evaluate its efficacy in a large population.

## 2 METHODS

### 2.1 Study design

**FIGURE 1:**
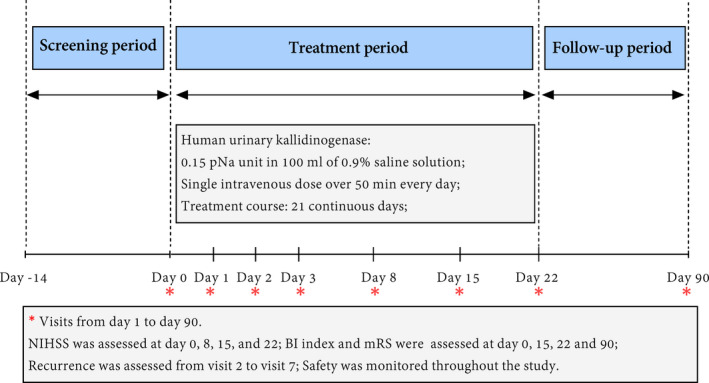
Study profile

In this single‐arm, multicenter, phase IV study (NCT02562183; Figure 1), patients were recruited from 65 centers in China between August 2015 and June 2020. The study was conducted in accordance with Declaration of Helsinki and International Conference on Harmonisation‐Good Clinical Practice guidelines. The study protocol was approved by the institutional review board (Ethical Approval Number: HS‐917). Written informed consent was obtained from all patients or their legal surrogates.

### 2.2 Patients eligibility

Patients aged 18–80 years were eligible if they had a clinical diagnosis of anterior circulation AIS with an onset within the previous 48 h. Details of diagnostic criteria for AIS have been described in the published protocol.^16^ Briefly, they had to be the first onset or had a history of ischemic stroke but without serious sequelae, with a modified Rankin Scale (mRS) score of 0–2 and a National Institute of Health Stroke Scale (NIHSS) score of 6–25.

We excluded patients with clinically suspected or confirmed intracranial hemorrhagic disease by head computed tomography (CT), transient ischemic attacks (TIA), or patients who previously underwent or to undergo thrombolysis, interventional therapy, and stenting. Patients who had previously received angiotensin‐converting enzyme inhibitors (ACEI) but within five half‐lives, or who require ACEI therapy after enrollment were ineligible. Patients were also ineligible if they are allergic or intolerant to HUK. Complete eligibility criteria are listed in the published protocol.^16^

### 2.3 Procedures

Patients were scheduled to receive HUK (0.15 pNa unit) as an intravenous infusion. HUK was administered as a single intravenous dose over 50 min every day for 14–21 continuous days according to clinical practice, until unacceptable AEs, consent withdrawal, or investigators’ decision. Patients who experienced treatment‐related AEs should pause their treatment and receive positive treatment, or withdrawal from the study. All patients were allowed to receive antiplatelet, anticoagulant, defibrase, and neuroprotective agents according to the guideline for AIS. After the completion of treatment, all patients were followed up to day 90. During the 90‐day follow‐up, the safety was monitored by a follow‐up telephone call. HUK (Techpool BioPharma Co. Ltd) was provided as the sterile solution and diluted in 0.9% saline solution before infusion.

### 2.4 Endpoints and assessments

The primary endpoint was the overall incidence of treatment‐emergent AEs throughout the study. Safety and tolerability were evaluated through AEs, vital signs, laboratory measurements (including urinalysis, hematology, and blood chemistry), electrocardiography, and neuroimaging data (head CT or magnetic resonance imaging) during infusion, the first 24 h post‐infusion, and at scheduled visits, including day 1, 2, 3, 8, 15, 22 or discharge, and 90‐day follow‐up. AEs were assessed using Medical Dictionary for Regulatory Activities (MedDRA) and graded as mild, moderate, and severe in severity. AEs were considered as serious if they resulted in any following conditions: death, a life‐threatening AEs, a congenital anomaly/birth defect, a persistent or significant incapacity or organ damages, in‐patient hospitalization or prolongation of existing hospitalization, a significant medical event that require intervention. The severity of AEs and the relationship to the intervention were determined by investigators. Drug‐related AEs were defined as AEs that investigators classified as possibly, probably, or definitely related to investigational drugs. Pre‐specified AEs of special interest (AESI) were identified using MedDRA preferred terms and selected as BP reduction, symptomatic intracranial hemorrhage (sICH), and abnormal renal/liver function. BP reduction was defined as BP < 90/60 mmHg, or a decrease in sitting BP accompanied by clinical symptoms (dizziness, etc.). BP was measured at scheduled time points during the treatment (day 1–3: 5, 10, 15, 30, 50, and 80 min before and after injection, respectively; day 8, day 15, and the end of the treatment: before and after injection). Abnormal renal/liver function refers to aspartate aminotransferase/alanine aminotransferase >1.5 × upper normal limit (UNL) or serum creatinine >1.0 × UNL. The sICH was defined as hemorrhagic transformation accompanied by neurological deterioration (increase in NIHSS score of at least 4).

Secondary endpoints included the functional improvement measured with NIHSS score, activities of daily living measured with Barthel index (BI), mRS score, and stroke recurrent rate. All patients underwent clinical assessment at various times (as indicated in Figure 1) throughout the study, including at baseline, on day 1, 2, 3, 8, 15, 22 or discharge, and 90‐day follow‐up. Examiners were trained and certified in the use of NIHSS (range 0–42; higher score indicating greater stroke severity), BI (range 0–100; 0 = complete dependence, 100 = independence), and mRS examination (range 0–5; 0 = no residual symptoms, 5 = bedridden and needs constant attention). In this study, patients who died during the study were assigned a mRS score of 6 and a BI score of 0. Recurrence was defined as neuroimaging‐proven AIS or TIA and assessed at all visits from the first 24 h post‐infusion to 90‐day follow‐up. When patients were unable to attend a follow‐up visit, scores were assessed by telephone calls.

### 2.5 Statistical analysis

Sample size calculation was based on the incidence of pre‐specified AESI. Assuming a 1.2% incidence of pre‐specified AESI,^17^ we calculated that a sample size of 1822 patients was expected to ensure a maximum 95% confidence interval width of 1% at a two‐sided significance level of 0.05. Expecting a proportion of patients lost to follow up of 20%, the total required sample size was estimated to be 2186 patients. Due to the COVID‐19 pandemic, an obvious decline in patient enrollment occurred, and thus, the study was prematurely terminated after 1208 patients had been enrolled.

Safety was assessed in patients who received at least one dose of the investigational drug. Data of AEs were summarized descriptively. Efficacy analyses were based on the intention‐to‐treat principle. Efficacy endpoints were analyzed, respectively, in full analysis set (FAS) and per‐protocol set (PPS). FAS was defined as patients who received at least one dose of the investigational drug and completed at least one clinical assessment. PPS was defined as patients who completed the treatment and had the absence of any major protocol violations. Standard summary statistics were calculated for all variables as appropriate to the data type. The Kolmogorov‐Smirnov test was performed for normality test. Statistical analysis was performed using paired t‐test for normally distributed continuous data and the Wilcoxon rank test for ordinal or nonparametric data. Incidence densities of AEs were calculated as the ratio of total patients who experienced AEs and total patient‐years of exposure. Risk factors for the occurrence of drug‐related BP reduction were analyzed using a stepwise logistic regression model and assessed by odds ratio (OR). Potential confounding factors were chosen on the basis of clinical importance, clinical experience, their association with stroke outcomes, and the results of analysis of sociodemographic and other characteristics of the sample. Statistical significance was set at *p* < 0.05 and analyzed using SAS software (version 9.4; SAS Institute Inc.).

## 3 RESULTS

### 3.1 Baseline characteristics

**FIGURE 2:**
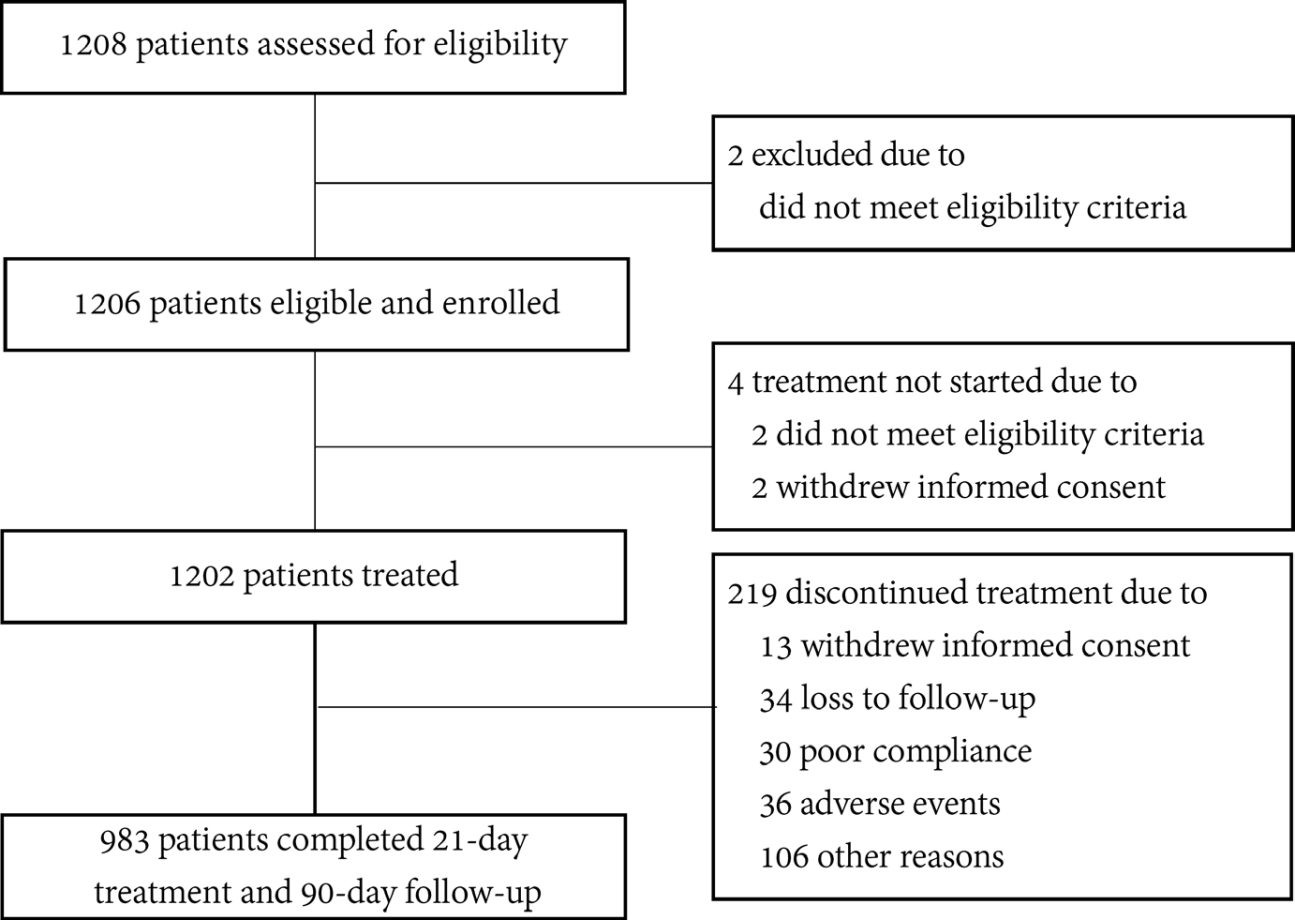
Patient flow diagram

A total of 1208 patients with AIS were screened from 65 centers in China between August 2015 and September 2020. Among them, 1206 were eligible and recruited (Figure 2). Of 1206 eligible patients, 4 patients did not receive the HUK infusion because of consent withdrawal (*n* = 2) and delayed recognition that patients were ineligible (*n* = 2). 983 (81.5%) patients completed the 21‐day treatment and 90‐day follow‐up assessment. The reasons for not completing the treatment included the withdrawal of consent (*n* = 13), loss to follow up (*n* = 34), poor compliance (*n* = 30), AEs (*n* = 36), or other factors (such as investigators' decision, n=106). Finally, 1199 patients were included in FAS and 677 in PPS. Baseline characteristics of 1202 patients who received treatment were listed in Table 1. The mean age of enrolled patients was 61.34 ± 10.67 years; of these, 68.05% were male. The majority of patients (92.06%) were independent pre‐stroke (mRS, 0–1).

**TABLE 1:** Baseline characteristics

Characteristics	Patients (*n* = 1202)
Age, years	
Mean (SD)	61.34 (10.67)
Median (IQR)	62 (54–69)
Sex, no (%)	
Male	818 (68.05)
Female	384 (31.95)
Body mass index (kg/m^2^)	
Mean (SD)	24.19 (3.15)
Median (IQR)	24.01 (22.15–26.03)
Smoking, no (%)	520 (43.30)
Drinking, no (%)	216 (17.99)
Time from stroke onset to treatment, h	
Mean (SD)	29.78 (13.20)
Median (IQR)	30.5 (20.8–40.3)
Previous stroke, no (%)	326 (27.12)
Independent pre‐stroke (mRS 0–1), no (%)	1101 (92.06)
NIHSS score	
Mean (SD)	8.32 (3.35)
Median (IQR)	7 (6–9)
BI score	
Mean (SD)	52.45 (24.66)
Median (IQR)	55 (30–70)
Stroke subtype based on TOAST, no (%)	
Atherothrombotic	808 (67.33)
Cardioembolic	9 (0.75)
Lacunar	317 (26.42)
Other/not differentiated	12 (1.00)
Unknown	54 (4.50)
Systolic blood pressure (mm Hg)	
Mean (SD)	147.74 (20.45)
Median (IQR)	148 (132.5–161)
Diastolic blood pressure (mm Hg)	
Mean (SD)	84.95 (12.93)
Median (IQR)	85 (76~94)
Concomitant disease, no (%)	
Cerebral hemorrhage	2 (0.17)
Hypertension	797 (66.31)
Diabetes mellitus	367 (30.53)
Hyperlipidemia	179 (14.89)
Coronary heart disease	163 (11.20)

Note

Data were expressed as mean (SD), median (interquartile range), or number (%).

Abbreviations: BI, Barthel index; h, hour; IQR, interquartile range; mRS, modified Rankin scale; NIHSS, National Institute of Health Stroke Scale; SD, standard deviation; TOAST, Trial of ORG 10172 in Acute Stroke Treatment.

### 3.2 Safety analysis

#### 3.2.1 Treatment‐emergent AEs

The overall incidence of treatment‐emergent AEs was 55.99% (95% CI, 53.18%–58.8%) in patients who received treatment (Table 2), with a median of 2.2 events per patient and an incidence density of 2.67 patients per 100 patient‐years. Frequently reported AEs included BP reduction (11.90%), constipation (9.73%), fever (3.58%), and elevated transaminase (3.49%). A total of 513 (42.68%) patients experienced unexpected AEs, such as constipation (8.49%), hyperhomocysteinemia (3.00%), and pulmonary infection (3.00%). Besides, 41 (3.41%) patients discontinued or withdrew because of treatment‐emergent AEs.

**TABLE 2:** Adverse events by MedDRA preferred term

	Number of events	Number of patients (*n* = 1202), *n* (%)
Any AEs	1483	673 (55.99)
Serious AEs	33	29 (2.41)
Unexpected AEs^a^	985	513 (42.68)
Any AEs leading to withdrawal	52	41 (3.41)
Most frequent AEs (≥3% incidence)^b^		
Blood pressure reduction^c^	183	143 (11.90)
Constipation	120	117(9.73)
Elevated transaminase	42	42 (3.49)
Hyperhomocysteinemia	37	37 (3.08)
Pulmonary infection	39	39 (3.24)
Fever	45	43 (3.58)
Drug‐related AEs^d^	240	186 (15.47)
Serious drug‐related AEs	7	7 (0.58)
Unexpected drug‐related AEs	67	58 (4.83)
Drug‐related AEs leading to withdrawal	27	19 (1.58)
All pre‐specified AESI	323	261 (21.71)
Blood pressure reduction^c^	183	143 (11.90)
Abnormal renal/liver function	132	126 (10.48)
sICH	8	8 (0.67)
Serious AESI	6	6 (0.50)
AESI leading to withdrawal	13	13 (1.08)
Drug‐related AESI	176	148 (12.31)
Drug‐related blood pressure reduction	120	100 (8.32)
Drug‐related abnormal renal/liver function	49	49 (4.08)
Drug‐related sICH	7	7 (0.58)
Serious drug‐related AESI	4	4 (0.33)
Drug‐related AESI leading to withdrawal	12	12 (0.33)

Note

Data were expressed as number (%).

Abbreviations: AEs, adverse events; AESI, adverse events of special interest; MedDRA, Medical Dictionary for Regulatory Activities; sICH, symptomatic intracranial hemorrhage.

^a^ Unexpected AEs are defined as those involving “any adverse drug experience that is not listed in the current labeling for the drug product.”

^b^ The events included are the most frequent AEs at an incidence of ≥3%.

^c^ Blood pressure reduction is both the most frequent AE and the pre‐specified AESI.

^d^ Drug‐related AEs were defined as those with a probable, possible or definite causality.

The majority of AEs were graded as mild (79.23%), 18.00% as moderate, and 2.77% as severe AEs. Serious AEs were reported in 2.41% (29/1202) of patients. Cerebral infarction (1.66%) and cerebral hemorrhage (0.42%) were the most frequently reported serious AEs. Only stroke recurrence (1 patient) and hypotensive shock (1) in serious severity were determined to be related to the investigational drug but completely relieved after symptomatic treatment.

AEs suspected to be drug‐related based on investigator assessment were recorded in 15.47% of 1202 patients, among which serious AEs were reported by 7 (0.58%) patients, unexpected AEs 58 (4.83%) patients, and AEs leading to withdrawal 19 (1.58%) patients. The most common drug‐related AEs were BP reduction (8.32%), followed by abnormal liver function (3.99%).

During the 21‐day treatment period and 90‐day follow‐up assessment, 5 (0.42%) deaths occurred. No death was thought to be related to study medication as determined by investigators. The adjudicated causes of death were cerebral infarction progression (1), aortic dissection (1), respiratory failure (1), and primary bronchogenic carcinoma (1). One death occurred outside the hospital due to an unknown cause, and the cause of death was judged as AEs by the investigator. No notable changes were observed in other vital signs, laboratory measurements, and electrocardiography.

#### 3.2.2 Pre‐specified AESI

The pre‐specified AESI were summarized in Table 2. A total of 323 pre‐specified AESI were reported in 261 (21.71%; 95% CI, 19.38%–24.04%) patients, with a median of 1.24 events per patient and an incidence density of 0.58 patients per 100 patient‐years. Of pre‐specified AESI, BP reduction (11.90%) was most commonly reported. 120 events in 100 patients were considered to be possibly related to investigational drug, but the majority were transient diastolic BP reduction. Notably, of these patients, only one patient experienced severe BP reduction but resolved the same day. The majority (136/143, 95.10%) of BP reduction was mild and expected. Abnormal renal/liver function was reported in 10.48% of patients, including 83 mild cases, 42 moderate, and 1 severe, and the events in 63.5% of these patients were determined to be unrelated to study therapy. Eight sICH events were reported in eight (0.67%) patients. Among them, five events were severe in severity but no event was fatal.

13 pre‐specified AESI events resulted in withdrawal, and 12 were considered to be possibly drug‐related. Six serious pre‐specified AESI were reported in six (0.50%) patients, among which four were determined to be possibly related to investigational drug, including cerebral hemorrhage (2), hemorrhagic infarct (1), and hypotensive shock (1). The hypotensive shock occurred due to the relatively fast infusion rate (<50 min).

#### 3.2.3 Risk factors for drug‐related BP reduction (pre‐specified AESI)

**FIGURE 3:**
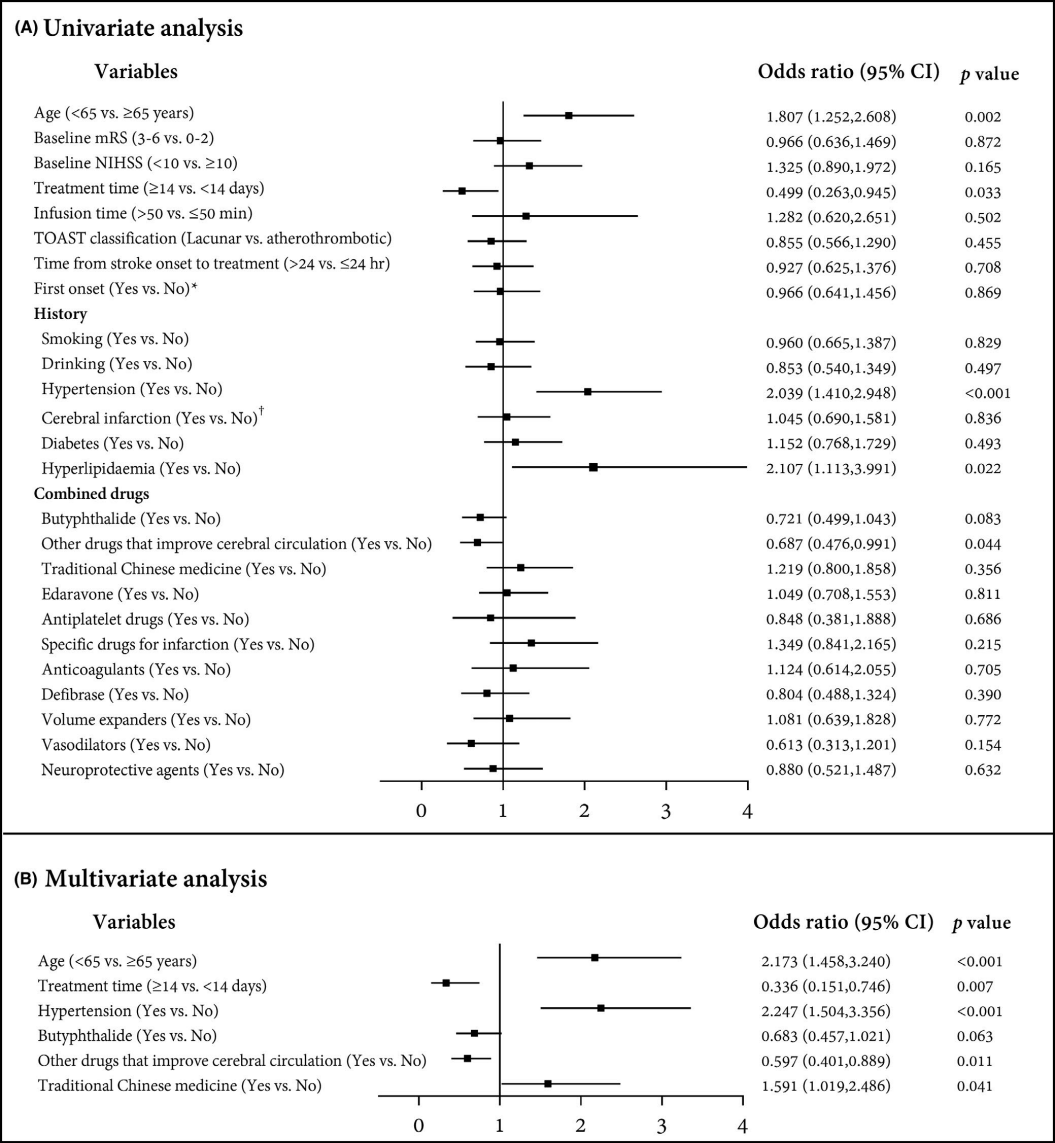
Forest plot of the logistic regression analysis. (A) Univariate analysis; (B) Multivariate analysis. * The “first onset” included the patients who were admitted to hospital admission for a first‐onset ischemic stroke but had no previous history of cerebral infarction. ^†^Cerebral infarction refers to the previous history of disease, including patients who had a cerebral infarction previously

Multiple regression analysis showed that the older age (OR = 2.173; 95% CI, 1.458–3.240; *p* < 0.001) and longer treatment time (OR = 0.336; 95% CI, 0.151–0.746; *p* = 0.007) were associated with a higher risk for drug‐related BP reduction, after adjustment for smoking, drinking, diabetes, hyperlipidemia, previous cerebral infarction, time from stroke onset to HUK treatment, baseline NIHSS, baseline mRS, and the previous use of anticoagulant or antiplatelet agents (Figure 3). Conversely, hypertension (OR = 2.247; 95% CI, 1.504–3.356; *p* < 0.001) was related to a lower risk for this event (Figure 3). Besides, the combination of traditional Chinese medicine (OR = 1.591; 95% CI, 1.019–2.486; *p* = 0.041) or other drugs that improve cerebral circulation (OR = 0.597; 95% CI, 0.401–0.889; *p* = 0.011) were also identified to be independently associated with the occurrence of drug‐related BP reduction (Figure 3). However, the association between infusion rate and this event was not identified (*p* > 0.05).

### 3.3 Efficacy

**FIGURE 4:**
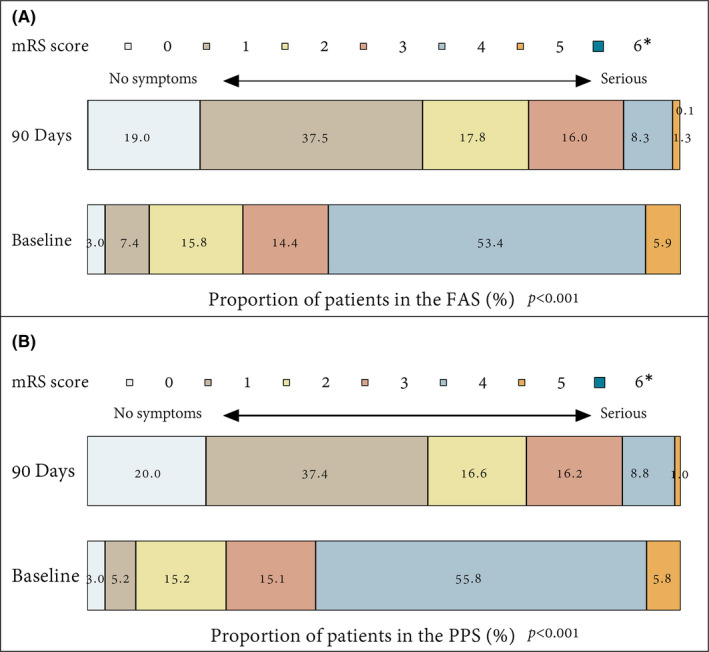
Distribution of mRS score at baseline and 90 days in the FAS and PPS population. Data were expressed as number (%). * Death was assigned a mRS score of 6 points. FAS, full analysis set; mRS, modified Rankin Scale; PPS, per‐protocol set

There was a shift in the distribution of mRS scores in favor of HUK infusion (Figure 4). At the 90‐day assessment, favorable neurological outcome (mRS, 0–2) was seen in 74.4% of patients in the FAS population, while only 26.2% at baseline (*p* < 0.001; Figure 4A). The results of a sensitivity analysis using the PPS population supported the primary analysis (90‐day vs. baseline: 74.0% vs. 23.4%; *p* < 0.001; Figure 4B). In addition, the NIHSS scores demonstrated the favorable neurological outcomes from baseline (8.32 ± 3.52 [FAS]; 8.36 ± 3.02 [PPS]) to 22‐day assessment (4.05 ± 3.58 [FAS]; 3.85 ± 3.29 [PPS]), with improvement by at least 1 point (4.15 ± 2.66, *p* < 0.001 [FAS]; 4.31 ± 2.43, *p* < 0.001 [PPS]). With regard to the BI score, patients showed an improvement in activities of daily living as indicated by an increase in points from baseline (52.45 ± 24.66 [FAS]; 51.62 ± 23.49 [PPS]) to 90‐day assessment (84.26 ± 21.2 [FAS]; 84.64 ± 20.96 [PPS]), with statistically significant differences (both *p* < 0.001). The changes in NIHSS and BI scores were shown in Figure S1. Besides, imaging examination during the 90‐day assessment identified 30 recurrent strokes, resulting in a cumulative recurrence rate of 2.50% in the FAS population (Figure S2A). Similar results were observed in the PPS population, with a cumulative stroke recurrence rate of 2.51% (Figure S2B). Overall, the data of efficacy endpoints between FAS and PPS were generally identical, confirming robustness of the data.

## 4 DISCUSSION

This multicenter, phase IV RESK study has provided the first detailed analysis of safety with HUK, as well as additional data on its efficacy in the large population with AIS. The study reported the favorable and acceptable safety profile for HUK, with a low incidence of serious AEs and no clinically significant risk. We also confirmed its clinical efficacy in a large population, presenting with significant neurological and functional improvements in patients with AIS. More importantly, we identified several risk factors for the occurrence of drug‐related BP reduction, providing high‐level evidence for its safety in clinical application.

In this study, the overall incidence of AEs was 55.99%; however, serious AEs only occurred in 29 (2.41%) patients, indicating a favorable safety profile for HUK. Theoretically, the incidence of AEs was relatively higher when compared with the previous trial with an estimate of 12.4%.^18^ Actually, our study was conducted in a real‐world setting without strict restriction on eligibility criteria (such as concomitant therapy, weight, and height), and thus, differences in the patient background may be responsible for the high incidence of AEs. Besides, in our study, any abnormal laboratory measurements or uncomfortableness reported by patients, neither with clinical significance or not, were recorded as AEs to make the safety assessed fully, which might be another cause of its high incidence. Notably, the incidence of serious AEs (2.41%) was similar to those in previous studies (1.2%).^11^, ^18^ In contrast, 43% of patients in the ReMEDy trial experienced serious AEs after a recombinant HUK (DM199) therapy.^19^ Besides, here, study withdrawals (3.41%) or death (1 patient) due to AEs were infrequent, with only a few occurrences. Reported AEs were generally mild, tolerable, and controllable, which resolved soon with symptomatic treatment, suggesting the acceptable tolerability profile of HUK. In fact, the safety profile observed here was consistent with that expected from patients receiving HUK, as reported in other studies.^18^, ^20^ Similar to those studies, BP reduction and fever were the most common AEs after HUK infusion. Thus, these events, especially BP reduction, should be alert and focused on in clinical practice. However, a small number (15.47%) of patients experienced drug‐related AEs, and among them, only 2.82% (incidence of 1.58%) were definitely drug‐related, which was similar to the results of DM199.^21^ Unexpectedly, the study reported 3 unexpected events with high frequency. However, no severe unexpected AEs occurred. Generally, HUK has an acceptable safety and tolerability profile from a safety perspective.

The occurrence of pre‐specified AESI was not frequent, except BP reduction. Among them, the incidence of sICH (0.67%) after HUK infusion was comparably lower than that after other therapies, such as alteplase (5.8%)^22^ and mechanical thrombectomy (1.5%).^23^ Although five sICH events were severe in severity, no event was fatal. BP reduction caused by HUK infusion is regarded as an important concern regarding the safety of HUK. Although the incidence of BP reduction reported here appeared to be relatively higher than those (1.5%–5.1%) in previous studies,^15^ only seven events were considered to be definitely drug‐related. Moreover, the majority were transient diastolic BP reduction, mild to moderate, clinically manageable, and disappeared in a short time. The drug‐related BP reduction occurred at manageable rates, although one of 1202 patients experienced a moderate hypotensive shock. On the one hand, the study had no restriction on the lower limit of the decrease in BP; as long as patients experienced clinical symptoms (dizziness, etc.) accompanied with a slight decrease in sitting BP, they were identified as AEs. Thus, the strict definition of BP reduction used here may explain part of its high incidence. As is well known, one principle of research is to minimize security risks in patients. Thus, from a security standpoint, the BP reduction was defined strictly in the present study. Actually, despite a proportion of patients who experienced BP reduction events, most patients did not achieve clinically meaningful reduction. On the other hand, the high incidence of BP reduction may be explained by a high proportion of elderly patients enrolled in our study, who are known to be at higher risk of hypotension and more sensitive to consequences of excessively low BP.^24^ This opinion was supported by logistic regression analysis, that patients aged above 65 years had a 2.173 times higher risk of drug‐related BP reduction. These data reminded us to individualize the treatment and management in this population. Additionally, treatment time of more than 14 days was the risk factor for drug‐related BP reduction, which may be related to the long‐time drug exposure. These data supported the idea that personalized treatment time options of HUK are of benefit to reduce the risk of BP reduction. In addition, the use of traditional Chinese medicine and other drugs that improve cerebral circulation in the base of HUK were identified to be associated with drug‐related BP reduction. Actually, the outstanding advantage of Chinese herbal formulas in antihypertensive effects has been widely accepted,^25^ which supported our finding. Conversely, the use of other drugs that improve cerebral circulation increased the additional drug exposure, leading to the increased risk of blood pressure reduction. However, given that the complexity and diversity of combination regimens, further verification is needed to determine how they influence BP reduction.

Interestingly, we found that hypertension patients had a lower risk for BP reduction. We speculated that the previous use of antihypertensive drugs in these patients may improve the endothelial function and self‐regulation of blood vessels,^26^ and hence, they are not sensitive to the effects of drugs that may cause BP reduction. Thus, the combination of antihypertensive therapy on the base of HUK may be unessential for AIS patients, even though hypertension patients. This finding was consistent with the recommendation in the Guidelines for the diagnosis and treatment of AIS in China.^27^ However, one patient experienced hypotensive shock due to the fast infusion rate, indicating a potential association between fast infusion rate and BP reduction. However, multivariate analysis did not highlight this association. In fact, only ten patients (0.8%) received HUK infusion within 50 min, and thus, the imbalance between the two subgroups may result in statistically non‐significant results. Nevertheless, the result cannot explain the clinical picture at present; accordingly, this association remains to be verified in further investigations. Thus, these factors, including age, treatment time, underlying hypertension, drugs combination, and infusion time, should be considered in the clinical application of HUK to minimize the risk of BP reduction. Overall, our study highlighted the potential factors associated with drug‐related BP reduction, providing recommendations to avoid the occurrence and consequences of BP reduction, especially for elderly patients.

In the light of efficacy analysis, results from our study are also encouraging, when compared with previous studies in AIS.^18^ Here, all secondary endpoints exhibited trends toward significant improvements, including the reduction in mean NIHSS and mRS scores as well as the increase in BI index, providing additional data on its efficacy in a large population. Besides, the favorable neurological outcome on mRS was seen in 74.4% of patients after HUK infusion, which was comparable with results of studies on other therapies, such as the MRCLEAN trial for intraarterial treatment (32.6%)^6^ and TTT‐AIS study for alteplase (53.4%).^28^ Additionally, it is well recognized that recurrent stroke (estimate range 2%–25%) is associated with increased disability and mortality.^29^ We herein evaluated the stroke recurrent rate. As result, the 90‐day cumulative incidence of recurrence was only 2.5% after HUK infusion, which was compared to 2.2% in the ReMEDy trial.^19^ Nevertheless, previous studies reported the stroke recurrence rate of 4.2%–7.4% in other cohorts.^30^, ^31^ In general, stroke recurrence events were un‐frequent in our study. Similar to our results, several meta‐analyses also demonstrated the effects of HUK, including decreasing stroke recurrence risk and marked neurological improvement.^20^ Regrettably, we cannot exclude the influence of the combined drugs or other factors on HUK efficacy because of the single‐arm study design. Therefore, we analyzed the effects of the usage of combined drugs on HUK efficacy using the subgroup analysis and logistic regression (Tables S1–S3). The results showed that the usage of other drugs (eg, other drugs that improve cerebral circulation and defibrase) is associated with the functional independence (mRS, 0–2), indicating that the combined drugs might not only affect the occurrence of AEs but also affect the clinical efficacy. However, most combined drugs were not identified to be associated with functional independence. The finding reinforces the necessity for the clinical consideration of these factors. Anyway, our findings serve to emphasize the clinical efficacy of HUK in the AIS treatment.

Our study still had some limitations. First, the sample size did not reach the intended target, which may have impacted the strength of the results. Actually, the hospitalization time for AIS patients was recommended within 7–14 days since 2016 according to the clinical practice. Thus, the majority of AIS patients were ineligible for our study that required 21‐day infusion, leading to slow enrollment. In addition, the slow enrollment of patients was aggravated due to the COVID‐19 pandemic, and thus, the study was prematurely terminated after 1208 patients had been enrolled. Although the study has a smaller sample size than planned, it is still deemed to be sufficient for the primary endpoint because the incidence of BP reduction has provided the statistical power of 85% in the interim analysis. Second, the uncontrolled, single‐arm, non‐randomized design is another potential criticism for our trial, which might not present the additional clinical benefits of HUK by comparing it to controls. However, from a design point of view, this design is more appropriate for the safety evaluation in a large population, and thus, our intent was to expose the maximum number of patients to treatment, so that safety (especially AESI events) could be assessed fully. Third, the patients were only recruited from the Chinese population, potentially limiting the generalizability of findings to the broader population. Finally, the cerebral blood flow of treated patients was not evaluated in the present study, which might not fully explained effects of HUK on the collateral circulation improvement. Although the function of HUK on cerebral blood perfusion has been previously demonstrated in the pre‐clinical study,^12^ its effects in patients remain unclear. Thus, due to the introduction of non‐invasive cerebral blood flow measurement in recent years, such as transcranial Doppler ultrasound and near‐infrared spectroscopy,^32^, ^33^ cerebral blood flow changes evaluation deserves further attention, and it would be the focus of future studies.

## 5 CONCLUSIONS

This phase IV study demonstrated that the intravenous HUK infusion in AIS patients had a favorable safety profile, with no serious safety concerns. The majority of AEs were unrelated to the study drug and had no clinical significance, further supporting the acceptable tolerability profile for HUK. Furthermore, we identified several risk factors for the BP reduction in special interest, providing guidance for the clinical application of HUK infusion in clinical practice. Additionally, improvements in functional outcomes were observed in patients, further confirming its clinical efficacy in a large population.

## CONFLICT OF INTEREST

All authors report no conflicts.

## Supporting information

Figure S1

Figure S2

Table S1‐S3

Supplementary Material

## Data Availability

The data that support the findings of this study are available from the corresponding author upon reasonable request.

## APPENDIX A

**The RESK Investigators**

Li‐Ying Cui, Peking Union Medical College Hospital, Chinese Academy of Medical Sciences, Beijing (Study Chair and Principal Investigator; 11); Jun Ni and Ming Yao, Peking Union Medical College Hospital, Chinese Academy of Medical Sciences, Beijing (Principal Investigator); Run‐Hui Li, Central Hospital affiliated to Shenyang Medical College, Shenyang (63); Li‐Hong Zhao, Dandong People's Hospital, Dandong (62); Li‐Hua Wang, The Second Affiliated Hospital of Harbin Medical University, Harbin (56); Jia‐Chun Wang, No. 1 Hospital of Harbin, Harbin (51); Ming Yu, The Affiliated Hospital of Jiangsu University, Zhenjiang (46); Yan‐Lei Hao, Affiliated Hospital of Jining Medical College, Jining (38); Xin‐Ping Liu, Handan Central Hospital, Handan (36); Jie Lin, Handan First Hospital, Handan (36); Bao‐Lin Shi, Weifang People's Hospital, Weifang (36); Long‐Xuan Li, Shanghai Pudong New Area Gongli Hospital, Shanghai (36); Shi‐Ze Li, Zhengzhou Central Hospital, Zhengzhou (35); Chao‐Dong Zhang, Northeast International Hospital, Shenyang (35); Xiu‐E Wei, Xuzhou Mining Group General Hospital, Xuzhou (32); Ya‐Fen Wei, Heilongjiang Provincial Hospital, Harbin (32); Guang‐Hua Li, The Second Hospital of Harbin, Harbin (31); Ben‐Yan Luo, The First Affiliated Hospital, Zhejiang University School of Medicine, Hangzhou (31); Jian‐Jun Zhang, Baoji Central Hospital, Baoji (30); Li‐Yun Wang, Liaoyang Petrochemical General Hospital, Liaoyang (27); Ying‐Qi Zhou, The 411th Hospital of the Chinese People's Liberation Army, Shanghai (26); Xin‐Yi Li, Shanxi Bethune Hospital, Taiyuan (26); Gao‐Hua Li, Fushun Mining Bureau General Hospital, Fushun (26); Jun Chen, The First Hospital of Lanzhou University, Lanzhou (24); Yin‐Zhou Wang, Fujian Provincial Hospital, Fuzhou (22); Yan Wei, Harleeson International Peace Hospital, Shijiazhuang (21); Ji‐Hua Chen, First People's Hospital of Chenzhou, Chenzhou (20); Wei‐Min Liu, The First People's Hospital of Guiyang, Guiyang (20); Qing‐Ke Bai, Shanghai Pudong New Area People's Hospital, Shanghai (20); Jian‐Ping Niu, The Second Affiliated Hospital of Xiamen Medical College, Xiamen (19); Shi‐Xiang Kuang, The Second Affiliated Hospital of Guiyang College of Traditional Chinese Medicine, Guiyang (17); Mei Dong and Shi‐Ping Li, The Second Hospital of Hebei Medical University, Shijiazhuang (17); Xin‐Tong Liu, The Second People's Hospital of Guangdong Province, Guangzhou (16); Xiang Gao, The Affiliated Hospital of Ningbo University, Ningbo (16); Yan‐Song Li, The 436th Hospital of Chinese People's Liberation Army, Shenyang (16); Kai Wang, The 305th Hospital of Chinese People's Liberation Army, Beijing (14); Wei‐Min Xiao, Dongguan People's Hospital, Dongguan (12); Hai‐Qing Song, Xuanwu Hospital Capital Medical University, Beijing (12); Guo‐Hua Zhao, The Fourth Affiliated Hospital of Zhejiang University School of Medicine, Yiwu (11); Xing‐Yue Hu, Sir Run Run Shaw Hospital Affiliated to Zhejiang University School of Medicine, Hangzhou (10); Guo‐Fang Chen, Xuzhou Central Hospital, Xuzhou (10); Bo Li, Beijing Hepingli Hospital, Beijing (10); Kan Ouyang, Hangzhou Red Cross Hospital, Hangzhou (10); Xin Wang, Zhongshan Hospital, Fudan University, Shanghai (8); Fu‐Ming Shi, Beijing Daxing District People's Hospital, Beijing (7); Yu‐Wu Zhao, The Sixth People's Hospital of Shanghai, Shanghai (7); Xiao‐Gang Li, Peking University Third Hospital, Beijing (6); Dan‐Hong Wu, The Fifth People's Hospital of Shanghai, Shanghai (6); Qiang Li, Central Hospital of Zibo, Zibo (6); Jing‐Bo Zhang, The Third People's Hospital of Dalian, Dalian (6); Guo‐En Yao, First affiliated Hospital of People's Liberation Army General Hospital, Beijing (5); Ding Qin, General Hospital of Beijing Jingmei Group, Beijing (5); Gang Li, Shanghai Eastern Hospital (South Hospital), Shanghai (4); Zheng‐Qi Lu, The Third Affiliated Hospital, Sun Yat‐sen University, Guangzhou (4); Qian Hou, Qinghai Provincial People's Hospital, Xining (3); Guang Huang, Fuxing Hospital, Capital Medical University, Beijing (3); Xiao‐Juan Lin, Fujian Hospital for the Aged, Fuzhou (3); Yi‐Ning Huang, Peking University First Hospital, Beijing (2); Jun Peng, Dongying People's Hospital, Dongying (2); Xiao‐Kun Qi, People's Liberation Army Navy General Hospital, Beijing (2); Jia‐Wei Wang, Beijing Tongren Hospital, Capital Medical University (South Hospital), Beijing (2); Qi‐Dong Chen, Yanda Hospital, Heibei Medical University, Sanhe (2); Qiang Ma, Zhongshan Hospital Affiliated to Dalian University, Dalian (1); Ping‐Yi Xu, The First Affiliated Hospital of Guangzhou Medical University, Guangzhou (1); Jia‐Wei Wang, Beijing Tongren Hospital, Capital Medical University, Beijing (1); Shu‐Yun Luo, Beijing Hospital of Integrated Chinese and Western Medicine, Beijing (1).
