# Supplementary figures and images for: Human urinary kallidinogenase in acute ischemic stroke: A single‐arm, multicenter, phase IV study (RESK study)
Source: CNS Neurosci Ther. 2021 Sep 12;27(12):1493–503. doi: 10.1111/cns.13724 (PMC8611767; doi:10.1111/cns.13724)

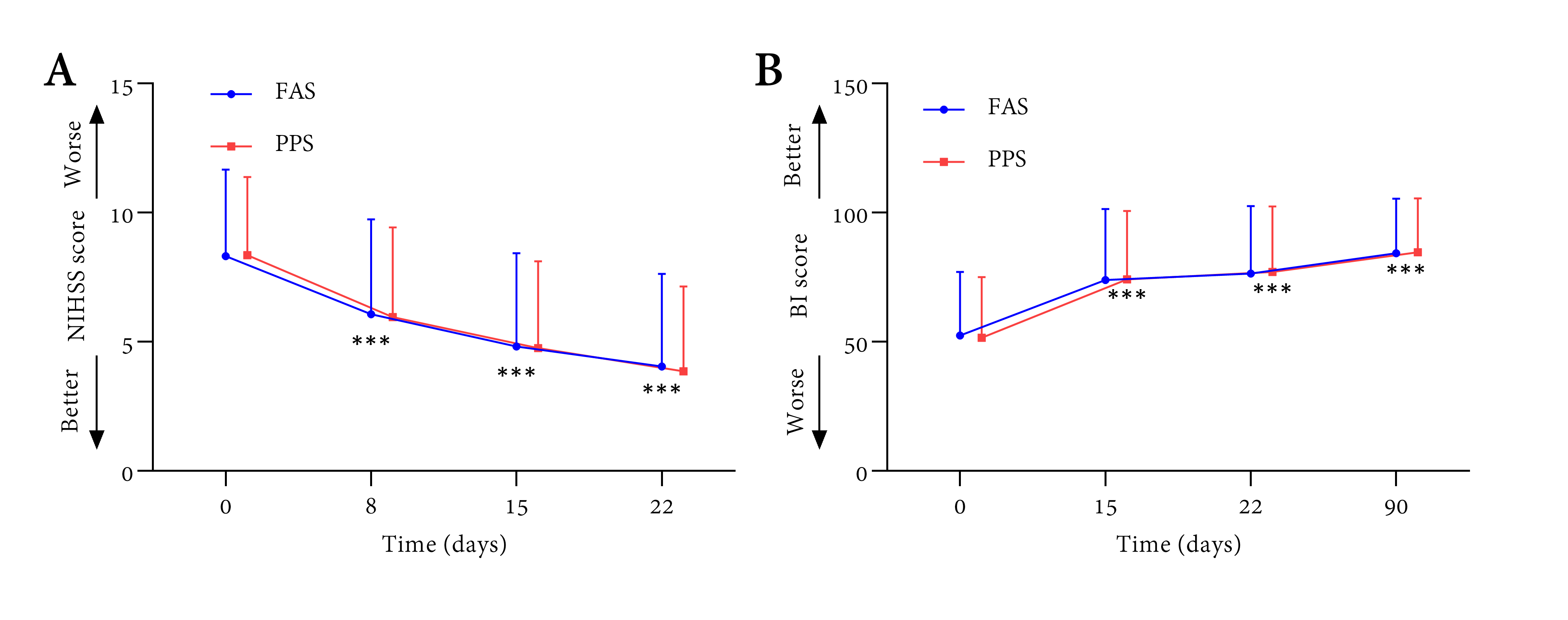

Supplement: Supplementary file 1 — Figure S1 [file CNS-27-1493-s002.tif]

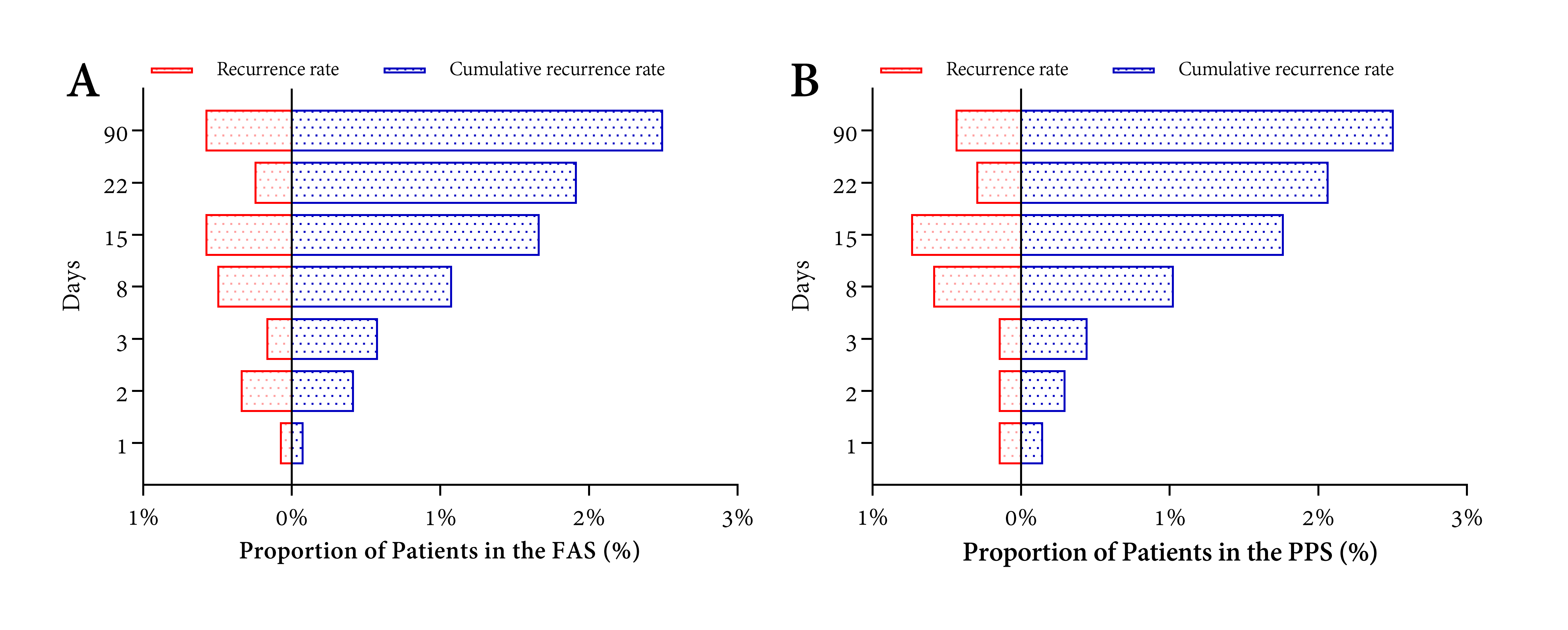

Supplement: Supplementary file 2 — Figure S2 [file CNS-27-1493-s003.tif]
