# Supplementary material for: Human urinary kallidinogenase in acute ischemic stroke: A single‐arm, multicenter, phase IV study (RESK study)
Source: CNS Neurosci Ther. 2021 Sep 12;27(12):1493–503. doi: 10.1111/cns.13724 (PMC8611767; doi:10.1111/cns.13724)
Supplement: Supplementary file 3 — Table S1‐S3 [file CNS-27-1493-s004.docx]

**Table S1. Subgroup analyses: proportions of patients achieved independent outcome stratified by combined drugs in HUK-treated patients (FAS)**

| Factor | Subgroups (n) | mRS (0-2) * | mRS (3-6) | P value |
| --- | --- | --- | --- | --- |
| Edaravone | No (308) | 243 (78.90%) | 65 (21.10%) | 0.066 |
|  | Yes (729) | 528 (72.43%) | 201 (27.57%) |  |
| Antiplatelet drugs | No (57) | 45 (78.95%) | 12 (21.05%) | 0.495 |
|  | Yes (980) | 726 (74.08%) | 254 (25.92%) |  |
| Anticoagulants | No (925) | 688 (74.38%) | 237 (25.62%) | 0.637 |
|  | Yes (112) | 83 (74.11%) | 29 (25.89%) |  |
| Defibrase | No (884) | 654 (73.98%) | 230 (26.02%) | 0.182 |
|  | Yes (153) | 117 (76.47%) | 36 (23.53%) |  |
| Volume expanders | No (880) | 662 (75.23%) | 218 (24.77%) | 0.245 |
|  | Yes (157) | 109 (69.43%) | 48 (30.57%) |  |
| Vasodilators | No (980) | 732 (74.69%) | 248 (25.31%) | 0.485 |
|  | Yes (57) | 39 (68.42%) | 18 (31.58%) |  |
| Butyphthalide | No (664) | 506 (76.20%) | 158 (23.80%) | 0.248 |
|  | Yes (373) | 265 (71.05%) | 108 (28.95%) |  |
| Other drugs that improve cerebral circulation | No (621) | 484 (77.94%) | 137 (22.06%) | 0.005 |
|  | Yes (416) | 287 (68.99%) | 129 (31.01%) |  |
| Neuroprotective agents | No (147) | 117 (79.59%) | 30 (20.41%) | 0.240 |
|  | Yes (890) | 654 (73.48%) | 236 (26.52%) |  |
| Traditional Chinese medicine | No (215) | 162 (75.35%) | 53 (24.65%) | 0.893 |
|  | Yes (822) | 609 (74.09%) | 213 (25.91%) |  |
| Specific drugs for infarction | No (818) | 620 (75.79%) | 151 (68.95%) | 0.066 |
|  | Yes (219) | 198 (24.21%） | 68 (31.05%) |  |

* mRS (0-2) indicated a functional independence.

Data were analyzed by Cochran-Mantel-Haenszel (CMH) chi-square test

**Table S2. Subgroup analyses: proportions of patients achieved the good outcome stratified by combined drugs in HUK-treated patients (PPS)**

| Factor | Subgroups (n) | mRS (0-2) * | mRS (3-6) | P value |
| --- | --- | --- | --- | --- |
| Edaravone | No (191) | 150 (78.53%) | 41 (21.47%) | 0.143 |
|  | Yes (483) | 349 (72.26%) | 134 (27.74%) |  |
| Antiplatelet drugs | No (29) | 23 (79.31%) | 6(20.69%) | 0.581 |
|  | Yes (645) | 476 (73.80%) | 169 (26.20%) |  |
| Anticoagulants | No (595) | 440 (73.95%) | 155 (26.05%) | 0.689 |
|  | Yes (79) | 59 (74.68%) | 20(25.32%) |  |
| Defibrase | No (551) | 400 (72.60%) | 151 (27.40%) | 0.028 |
|  | Yes (123) | 99 (80.49%) | 24 (19.51%) |  |
| Volume expanders | No (564) | 421(74.65%) | 143 (25.35%) | 0.599 |
|  | Yes (110) | 78 (70.91%) | 32 (29.09%) |  |
| Vasodilators | No (646) | 479 (74.15%) | 167 (25.85%) | 0.888 |
|  | Yes (28) | 20 (71.43%) | 8 (28.57%) |  |
| Butyphthalide | No (426) | 320 (75.12%) | 106 (24.88%) | 0.611 |
|  | Yes (248) | 179 (72.18%) | 69 (27.82%) |  |
| Other drugs that improve cerebral circulation | No (418) | 328 (78.47%) | 90 (21.53%) | 0.003 |
|  | Yes (581) | 171 (66.80%) | 85 (33.20%) |  |
| Neuroprotective agents | No (93) | 74 (79.57%) | 19 (20.43%) | 0.310 |
|  | Yes (581) | 425 (73.15%) | 156 (26.85%) |  |
| Traditional Chinese medicine | No (126) | 96 (76.19%) | 30 (23.81%) | 0.672 |
|  | Yes (548) | 403 (73.54%) | 145 (26.46%) |  |
| Specific drugs for infarction | No (524) | 396 (75.57%) | 128 (24.43%) | 0.133 |
|  | Yes (150) | 103 (68.67%) | 47 (31.33%) |  |

* mRS (0-2) indicated a functional independence.

Data were analyzed by Cochran-Mantel-Haenszel (CMH) chi-square test

**Table S3. Logistic regression: multivariate analysis of factors influencing the efficacy outcomes in HUK-treated patients**

| Variables | Multivariate analysis (FAS) | |
| --- | --- | --- |
|  | OR (95% CI) | p value |
| **FAS population** |  |  |
| Age (<65 vs. ≥65 years) | 0.682 (0.505, 0.921) | 0.0126 |
| Disease course (≥24 vs. <24 hours) | 1.407 (1.019, 1.944) | 0.0381 |
| Baseline mRS score (3-6 vs. 0-2) | 8.047 (4.669, 13.871) | <0.001 |
| Volume expanders (Yes vs. No) | 1.405 (0.946, 2.087) | 0.0923 |
| Other drugs that improve cerebral circulation (Yes vs. No) | 1.573 (1.167, 2.122) | 0.0307 |
| **PPS population** |  |  |
| Baseline mRS score (3-6 vs. 0-2) | 7.622 (3.787, 15.343) | <0.001 |
| Other drugs that improve cerebral circulation (Yes vs. No) | 1.756 (1.223, 2.522) | 0.0023 |

FAS=full analysis set; PPS=per-protocol set.
