# Supplementary material for: Human urinary kallidinogenase in acute ischemic stroke: A single‐arm, multicenter, phase IV study (RESK study)
Source: CNS Neurosci Ther. 2021 Sep 12;27(12):1493–503. doi: 10.1111/cns.13724 (PMC8611767; doi:10.1111/cns.13724)
Supplement: Supplementary file 4 — Supplementary Material [file CNS-27-1493-s001.docx]

## Supplementary materials

***Figure S1. Changes of secondary endpoints from baseline to assessment. (A) NIHSS score; (B) BI score.***

Data were expressed as mean and standard deviation.

NIHSS=National Institute of Health Stroke Scale; BI=Barthel index; FAS=full analysis set; PPS=per-protocol set. ***, *p*<0.001 vs. baseline (day 0).

***Figure S2. Recurrence rate of stroke during 90-day assessment in the FAS and PPS population.***

FAS=full analysis set; PPS=per-protocol set.

Recurrence was defined as neuroimaging-proven acute ischemic stroke or transient ischemic attacks. Data were expressed as number (%).

FAS=full analysis set; PPS=per-protocol set.
